# Hepatitis E Virus (HEV) Infection Among Pigs in Poland: An Assessment of the Prevalence on Commercial Swine Farms

**DOI:** 10.1155/tbed/7140750

**Published:** 2026-06-25

**Authors:** Iwona Kozyra, Justyna Joniec-Wiechetek, Jacek Żmudzki, Artur Rzeżutka

**Affiliations:** ^1^ Department of Microbiology of Food and Feed, National Veterinary Research Institute, Al. Partyzantów 57, Puławy, 24-100, Poland, piwet.pulawy.pl; ^2^ Department of Bacteriology and Bacterial Animal Diseases, National Veterinary Research Institute, Al. Partyzantów 57, 24-100, Puławy, Poland, piwet.pulawy.pl

**Keywords:** detection, fattening pigs, HEV, infection prevalence, subtype identification

## Abstract

Hepatitis E is a zoonotic infection related to consumption of virus‐containing food of pig origin. Pigs are a natural reservoir of hepatitis E virus (HEV), and infected animals can enter the food chain. The study evaluated the prevalence of HEV infections in pigs raised on 30 commercial pig farms in Poland. Six hundred pooled samples of pig faeces were collected from fatteners (440), gilts (69) and dry sows (91). For HEV detection a one‐step real‐time reverse‐transcription PCR (real‐time RT‐PCR) method with an incorporated target‐specific internal amplification control was used. Subtype identification of the detected HEV strains was based on amplification and phylogenetic analysis of the open reading frame (ORF) 2 region in the virus genome. Viral RNA was detected in 161 (26.8%; 95% confidence interval [CI]: 23.3–30.6) samples of pooled pig faeces. Fattener pigs were by far the most prevalently infected (35.9%; 95% CI: 31.4–40.6; odds ratio [OR] = 18.7, 95% CI: 4.9–159.6), and gilts were markedly less so (2.9%; 95% CI: 0.3–10.1; OR = 0.05, 95% CI: 0.006–0.20). HEV‐positive pigs were raised on fattening units (OR = 1.6, 95% CI: 1.1–2.4) and farrow‐to‐finish farms (OR = 0.6, 95% CI: 0.4–0.9) without any differences in prevalence of infections associated with farm type (*χ* ^2^ = 2.4, *p* < 0.121). Phylogenetic analysis of the detected pig HEV strains affiliated them to the HEV‐3c, 3i, 3e and 3f subtypes. Strains of the HEV‐3i subtype dominated on farrow‐to‐finish farms (*χ* ^2^ = 33.4, *p* < 0.001), while 3f strains did so on fattening farms (*χ* ^2^ = 19.1, *p* < 0.001). The results evidence HEV mainly being prevalent in fattening pigs at the age of 5–6 months. Virus presence in these animals could pose a food‐related risk of HEV infection when they reach slaughter age.

## 1. Introduction

Hepatitis E virus (HEV) was first detected in humans in the early 1980s during an epidemic of viral hepatitis in humans in India [1–3]. However, it was not until 1997 that the first HEV infections in farm animals were detected in the United States of America [4]. These infections were in pigs. In subsequent years, the virus was also detected in other livestock species besides pigs, as well as in wildlife and in birds [5]. HEV strains detected in pigs have been classified to the genus *Orthohepevirus*, family *Hepeviridae*, which also encompasses strains belonging to one out of seven virus genotypes [6]. Genotypes 1 and 2 were only found in humans, while genotypes 3 and 4 were detected in different animal species, including pigs [7, 8]. In Europe, mainly HEV‐3 strains circulate in pigs, although the introduction of HEV‐4 has been detected in fattening pigs in Belgium [9]. Of note is that genotype 4 is predominant in Asia, and the majority of virus strains have been detected in China so far [10]. Analysing the similarities and differences in the genome structure of individual virus strains within HEV‐3, 10 virus subtypes (a–c, e, f and h–m) have been established. However, not all detected virus sequences originated from pig and wild boar, and their subtypes could be identified and subsequently assigned to any virus sub‐cluster due to their low phylogenetic relationship [6, 10]. By examining the frequency of HEV‐3 subtypes detection, strains of 3a, 3e and 3f showed worldwide distribution. Contrastingly, genotype 3c showed specific geographical location as it has been detected in pigs and wild boars from Central Europe [10]. Wild boars and pigs are the main reservoirs of the virus for humans [11]. The majority of infections are observed in pigs, which become infected via the faecal‐oral route [12–14]. Piglets are particularly susceptible to infection when they lose colostrum‐derived passive immunity [15, 16]. The presence of specific anti‐HEV antibodies is mainly detected in animals aged between 6 and 22 weeks, with the highest levels observed in fattening pigs over 13 weeks of age [17]. In Europe, there are differences observed in the prevalence of infections in pigs from the same production groups. For example, a high 94.3% seroprevalence has been observed in fattening pigs in Spain, while it was 8.3% in fatteners bred in Corcica [18, 19]. Differences in virus seroprevalence can also result from the age of the tested pigs being at different stages of fattening. Usually, high seroprevalence values are found in sows, i.e., the oldest (>12 months of age) group of pigs present on the farm. However, the presence of specific anti‐HEV antibodies in animals does not correlate with the presence of the virus antigen, which indicates active infection. Infections mainly affect both weaned piglets [20, 21] and fattening pigs [22–25], while they are sporadically reported in sows in Europe [17]. When analysing the infection pattern in different production groups of pigs, viral RNA was the most frequently detected in fattening pigs [20, 26, 27]. Serological studies confirmed the occurrence of HEV infections in pigs and wild boars in Poland 17 years after the first cases of pig infection were reported in the USA [28, 29], although the presence of zoonotic virus strains belonging to the 3c, 3i, 3e and 3f subtypes in Polish pigs has only been demonstrated recently [30]. It is noteworthy that HEV‐3 strains have been previously identified in pig livers and blood intended for consumption [31]. Among other livestock species than pigs being farmed in Poland, HEV‐3 infections have only been detected in rabbits [32]. In addition, the presence of HEV RNA was noted in wild animals such as wild boars, in which the 3i and 3a genotypes predominated [33, 34]. The asymptomatic nature of HEV infections in pigs, the presence of an animal HEV reservoir, and foodborne cases of human infection indicate the need for continuous monitoring of virus infections in pigs to limit their spread in this animal host. Virus detection in pigs, along with the identification of circulating virus genotypes, will expand the existing knowledge of HEV epidemiology in this animal species. The aims of the study were an evaluation of the prevalence of HEV infections in pigs raised on the different types of farm‐producing pigs in Poland and identifications of the virus subtypes causing them, as well as the analysis of their geographical and farm‐type‐related distribution pattern.

## 2. Materials and Methods

### 2.1. Pig Faecal Samples

**Figure 1:**
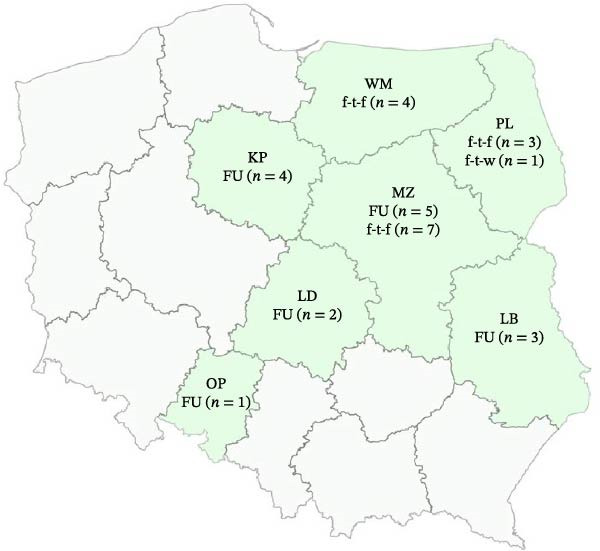
Geographical location of sampled farms in Poland. f‐t‐f, farrow‐to‐finish farm; f‐t‐w, farrow‐to‐wean farm; FU, fattening unit.

Six hundred pooled samples of pig faeces were collected from fattening (15), farrow‐to‐finish (14) and farrow‐to‐wean (1) farms with unknown histories of HEV infection. Convenience sampling was performed because not all major farms from the selected regions could be enrolled owing to African swine fever restrictions. Sampling was performed from September 2020 to June 2021. The farms were located in seven provinces in the central and eastern regions of Poland (Figure 1).

**Figure 2:**
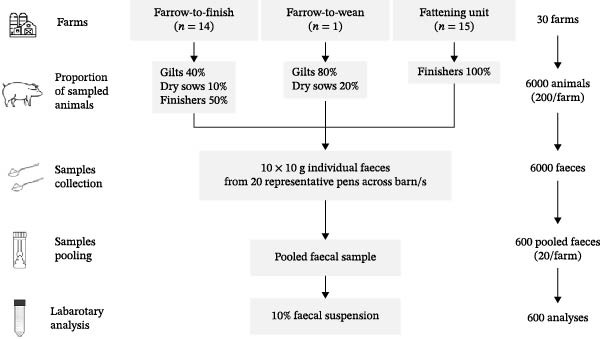
A diagram of faecal samples collection from pigs.

From each farm with a specific type of production, the particular groups of animals (fatteners/gilts/dry sows) were sampled at the following ratio: (a) farrow‐to‐finish farms—10 samples from fatteners (50%), 8 from gilts (40%) and 2 from dry sows (10%), (b) farrow‐to‐wean farms—16 samples from dry sows (80%) and 4 from gilts (20%) and (c) from fattening farms—20 (100%) fatteners faeces (Figure 2).

In the case of f‐t‐f farms, when the number of gilts was lower than expected due to the lack of sow replacement, the remaining number of samples was collected from dry sows. This sampling plan was originally devised for identification of the effective biosecurity measures for controlling HEV transmission on pig farms with unknown HEV status.

In this study, fatteners were animals aged 5–6 months, gilts were ~6 months of age before the first delivery of a litter of piglets, and dry sows were older than 12 months. Twenty pooled faecal samples were collected during a single visit at each farm. The sample pool was prepared by combining ~10 g portions of the freshly voided individual faeces of 10 animals. Faeces were collected from the pen’s floor into a sterile plastic pot using a disposable spoon. The sampling was performed cross‐sectionally, as animals housed in different pens representing particular barn sections were sampled. Each pooled sample originated from a single pen unless a pen contained less than six pigs, whereby it was collected from two other pens having pigs of the same age. Gloves and spoons were changed after the collection of each pooled sample. A total of 440 pooled faecal samples of fatteners, 91 faecal samples of dry sows and 69 faecal samples of gilts were analysed (Table 1). Testing of 20 pooled samples per farm provided sufficient sensitivity to detect at least one positive sample with a herd prevalence of ≥2% at the 95% confidence level [26, 30]. Upon arrival at the laboratory, the samples were stored at −20°C. They were kept in this condition until analysis.

**Table 1:** The number of sampled pigs and monitored farms across Polish provinces.

Province	Farm type	Number of farms	Number of 10‐animal pooled samples			Total
			From gilts	From dry sows	From fatteners	
Kujawy‐Pomerania (KP)	FU	4	0	0	80	80
Lubelskie (LB)	FU	3	0	0	60	60
Łódź (LD)	FU	2	0	0	40	40
Mazovia (MZ)	f‐t‐f	7	33	37	70	140
	FU	5	0	0	100	100
Opole (OP)	FU	1	0	0	20	20
Podlasie (PL)	f‐t‐w	1	4	16	0	20
	f‐t‐f	3	13	17	30	60
Warmia‐Masuria (WM)	f‐t‐f	4	19	21	40	80
Total	—	30	69	91	440	600

Abbreviations: f‐t‐f, farrow‐to‐finish farm; f‐t‐w, farrow‐to‐wean farm; FU, fattening unit/farm.

### 2.2. Farm Characteristics

In 2020, nearly 14 million pigs were being reared in Poland. Fattening pigs comprised 43.3%, and weaner pigs before transfer to fattening units made up 29.4% [35]. Faecal samples were collected on large commercial pig farms with farrow‐to‐finish and open farrow‐to‐wean production cycles and on fattening farms. The monitored farms were located in central, south‐central, north‐central, eastern and north‐eastern Poland. The farrow‐to‐finish farms housed from 70 to 1500 Danbred, Choice Genetics and PIC (Pig Improvement Company) sows, and the farrow‐to‐wean farm bred only Danbred sows. The fattening farms were stocked with 1 400–4 800 weaners of various breeds in every production cycle. The fattening period was about 13 weeks, and the animals were slaughtered at an age of 5.5 months. Pigs were provided with a commercial feed containing grain meal made on the site or soybean meal, both supplemented with minerals and vitamins. The farms have implemented internal and external biosecurity measures, that is, routine disinfection and rodent elimination, compliance with hygiene rules and routine husbandry training of personnel. The farrow‐to‐finish farms operated according to the all‐in/all‐out rule.

### 2.3. Sample Process Control Virus (SPCV)

Feline calicivirus (FCV) with a titre of 10^5.62^ TCID_50_/mL was used as a SPCV and was added to faecal supernatants obtained from their suspensions before analysis. This strain is in the virus collection of the Department of Microbiology of Food and Feed at the National Veterinary Research Institute in Poland. The preparation of the virus suspension in cell culture was as previously described by Rzeżutka [36].

### 2.4. Extraction of Viral RNA From Pig Faeces

A pooled faecal sample was thoroughly mixed, and a 100‐mg portion was added to 900 µL of PBS. The mixture was vortexed to obtain a 10% faecal suspension. The sample was clarified by centrifugation at 13,000 × *g* for 5 min and spiked with 10 µL of SPCV. Viral nucleic acids were isolated from 140 µL of faecal suspension using a QIAamp Viral RNA Mini Kit (Qiagen, Hilden, Germany) according to the manufacturer’s instructions. At this stage of sample analysis, positive (HEV‐containing faeces) and negative controls (reagents used for RNA extraction and water instead of the faecal sample) were included. The nucleic acids were eluted in DNase‐ and RNase‐free water in a final volume of 100 µL. The obtained nucleic acid extracts were assayed immediately or stored at −80°C.

### 2.5. HEV and SPCV Detection

Virus‐specific one‐step real‐time reverse‐transcription PCR (real‐time RT‐PCR) methods employing the RNA UltraSense One‐Step Quantitative RT‐PCR kit (Invitrogen, Basingstoke, UK) were used for the detection of HEV and SPCV. Briefly, HEV RNA detection was performed according to the protocol described by Maunula [37] using primers and a probe that allowed amplification of the HEV open reading frame (ORF) 3 genome fragment [38]. Molecular detection of viral RNA was monitored using the positive (HEV RNA) and negative (DNase‐ and RNase‐free water instead of the RNA sample) reaction controls, as well as an internal amplification control consisting of plasmid DNA containing sequences homologous to the primers used in the PCR [39]. The primers and probes along with the temperature‐time profile used for FCV detection were previously described by Lowther [40]. Only the reverse transcription step for cDNA synthesis was subjected to a minor modification, as it was shortened to 15 min at 50°C. The reactions were carried out on a 7500 Real‐Time PCR System (Applied Biosystems, Foster City, CA, USA). The recovery rate of process control virus from faecal samples was assessed by comparing the quantification cycle (Cq) value of FCV recovered from spiked faecal supernatants with the Cq value obtained from RNA derived from virus inoculum used to artificially spike the samples using the formula 2^−ΔCt^ × 100 [41]. Samples showing extraction recovery higher than 1% were considered acceptable, as stipulated in standardised methods for food‐borne virus detection [42].

### 2.6. Sequence and Phylogenetic Analysis of HEV Strains

Identification of the detected HEV strain subtypes and assessment of their phylogenetic relationships were based on the sequence analysis of a 493‐nt fragment of the ORF2 region in the HEV genome. Amplification was carried out by a two‐step nested RT‐PCR [43] using a SuperScript IV First‐Strand Synthesis System kit (Invitrogen, Vilnius, Lithuania). Purification and sequencing of PCR amplicons are described elsewhere [44]. Based on the consensus sequences of pig HEV strains, their genotypes were determined using the Hepatitis E Virus Genotyping Tool, v. 1.0 (https://www.rivm.nl/mpf/typingtool/hev/). The phylogenetic relationships of detected pig HEV strains were analysed by the maximum likelihood method with a Tamura‐Nei parameter model using MEGA 7.0 [45]. When several strains of a particular subtype exhibited 100% nucleotide sequence identity of the analysed gene fragment, only one strain representing a group of strains of a given subtype was selected for determination of its phylogenetic relatedness. The consensus nucleotide sequences of ORF2 fragments were compared to the full‐length or partial reference sequences of HEV‐3 strains [6]. The phylogenetic relationships were considered reliable when the bootstrap value was >70% for HEV strains clustered within the same subtype. The nucleotide sequences of the HEV strains were deposited in the GenBank database under Accession Numbers OQ686829–OQ686862.

### 2.7. Statistical Analysis

The prevalence of HEV infections in different groups of pigs (gilts, dry sows and fatteners) and on different farm types was estimated by the Clopper–Pearson method [46, 47]. Subsequently, Pearson’s chi‐squared (*χ* ^2^) test was employed to assess differences in the prevalence of infections between tested groups of animals and farm types. It was also used to determine the dominating HEV subtype in the pig population and on a particular farm type. Additionally, Fisher’s exact test was used with a subsequent estimation of the odds ratio (OR) values for the frequency of the virus occurrence in animals and different farm types. The calculations were performed using R software v. 4.1.1 [46] with the prevalence package used for the Clopper–Pearson method [47]. The adopted significance level was *p* = 0.05.

## 3. Results

### 3.1. Detection of HEV Infections in Pigs

**Figure 3:**
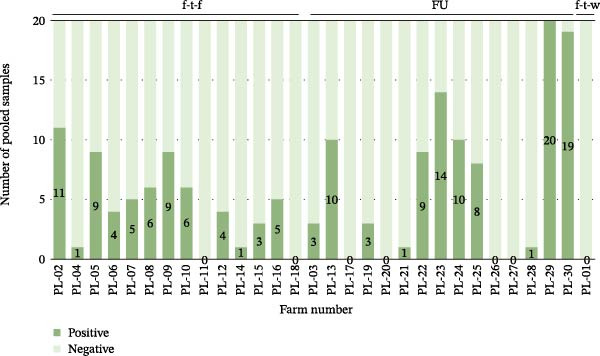
Distribution of positive pooled faecal samples detected in pig farms of particular production type. f‐t‐f, farrow‐to‐finish farm; f‐t‐w, farrow‐to‐wean farm; FU, fattening unit.

HEV RNA was detected in 161 out of 600 pooled pig faeces (26.8%; 95% confidence interval [CI]: 23.3–30.6) (Table 2). Nucleic acid extractions from faeces gave a mean logarithmic SPCV recovery rate of 93.2% (7.2%–100%). Proportionally, fattening pigs were observed to have the highest infection rate (35.9%; 95% CI: 31.4–40.6; OR = 18.7, 95% CI: 4.9–159.6), and gilts to be markedly less so (2.9%; 95% CI: 0.3–10.1; OR = 0.05, 95% CI: 0.006–0.20) (*χ* ^2^ = 19.4, *p* < 0.001). HEV‐positive pigs were raised on fattening units (fatteners; OR = 1.6, 95% CI: 1.1–2.4) and farrow‐to‐finish farms (fatteners and gilts; OR = 0.6, 95% CI: 0.4–0.9) with no statistically significant differences detected in prevalence of infections associated with farm type (*χ* ^2^ = 2.4, *p* < 0.121). Only one sample (1.3%; 95% CI: 0.03–7.2) of pooled faeces collected from dry sows from farrow‐to‐finish farms appeared to be virus positive. None of the tested gilts or dry sows from the farrow‐to‐wean unit shed the virus. Viral RNA was found on 11/14 (78.6%; 95% CI: 49.2–95.3) and 11/15 (73.3%; 95% CI: 44.9–92.2) of farrow‐to‐finish and fattening farms, respectively (Table 3). On HEV‐positive farrow‐to‐finish farms, the proportion of positive pooled samples per farm ranged from 5% (1 out of 20) to 55% (11 out of 20). However, there was only one farm having more than 50% virus‐positive pooled samples. On positive fattening units, the proportion of positive pools ranged from 5% (1 out of 20) to 100% (20 out of 20). On 5 units, virus was detected in at least half of the collected pooled samples, and on one farm, all samples appeared to be virus positive (Figure 3).

**Table 2:** HEV detection in pigs raised on different farm types.

Farm type	Number of samples							
	Fatteners		Gilts		Dry sows		Total	
	Positive/tested	Percentage (95% CI)	Positive/tested	Percentage (95% CI)	Positive/tested	Percentage (95% CI)	Positive/tested	Percentage (95% CI)
Fattening unit	97/300	32.3 (27.1–37.9)	0/0	—	0/0	—	97/300	32.3 (27.1 –37.9)
Farrow‐to‐finish	61/140	43.6 (35.2–52.2)	2/65	3.1 (0.4–10.7)	1/75	1.3 (0.03–7.2)	64/280	22.8 (18.1 –28.2)
Farrow‐to‐wean	0	—	0/4	—	0/16	—	0/20	—
Total	158/440	35.9 (31.4–40.6)	2/69	2.9 (0.3–10.1)	1/91	1.1 (0.02– 6.0)	161/600	26.8 (23.3–30.6)

**Table 3:** Farm‐level prevalence of HEV‐positive herds by farm type, number of positive pooled faecal samples and geographic distribution.

Farm type	Number of farms		Min–max number of positive pooled samples	Province (number of positive farms)
	Positive/tested	Percentage (95% CI)		
Fattening unit	11/15	73.3; 44.9–92.2	1–20	Kujawy‐Pomerania (2) Lublin (2) Łódź (2) Mazovia (4) Opole (1)
Farrow‐to‐finish	11/14	78.6; 49.2–95.3	1–11	Mazovia (4) Podlasie (3) Warmia‐Masuria (4)
Farrow‐to‐wean	0/1	—	—	—
Total	22/30	73.3; 54.1–87.7	1–20	Kujawy‐Pomerania (2) Lublin (2) Łódź (2) Mazovia (8) Opole (1) Podlasie (3) Warmia‐Masuria (4)

### 3.2. Sequence Analysis and Genotype Determination of Pig HEV Strains

The specific amplicons of the ORF2 region of the virus genome were obtained for 161 HEV strains. Subsequently, consensus sequences were generated for 126 strains. The successful identification of the virus subtype was achieved for 115 strains, whereas 11 strains were not assigned to any subtype group. The chromatograms of the remaining 35 nucleotide sequences were unreadable, or the sequence reads were only obtained from one DNA strand. HEV sequences submitted to the GenBank represented virus strains assigned to 3c (2 strains), 3f (18 strains), 3e (8 strains) and 3i (6 strains) subtype. They were considered as representative sequences for other virus strains of the same subtype, which circulated in pig farms of the particular production type. In each subtype group of strains, they shared a 100% nucleotide sequence similarity. Phylogenetic analysis of the ORF2 sequences of the detected pig HEV strains and their comparison to other HEV‐3 strains originating from humans, pigs and wild boars proved their affiliation to 3c, 3i, 3e and 3f virus subtypes.

### 3.3. Geographical and Farm‐Type Distribution of HEV Subtypes

Pigs were infected with HEV strains of the 3c, 3i, 3e and 3f subtypes (Table 4). The majority of detected strains were in the 3f subtype group (*χ* ^2^ = 4.2, *p* = 0.041). They were more often observed in the MZ and LD provinces than in other regions of the country; however, subtypes 3c, 3i and 3e were also detected on MZ farms. Strains from only two subtype groups were present on farms located in the PL (3i and 3f), WM (3i and 3e), KP (3c and 3f) and LB (3e and 3f) provinces. A single subtype circulating to the exclusion of the other three was only noted in the LD and OP provinces and was the 3f group. All detected virus subtypes were circulating in farrow‐to‐finish farms. With the exception of 3i strains, a similar pattern of genotype occurrence was also observed in fattening units. HEV‐3i strains dominated on farrow‐to‐finish farms (*χ* ^2^ = 33.4, *p* < 0.001), while 3f strains did so in fattening units (*χ* ^2^ = 19.1, *p* < 0.001). Virus strains not assigned to any subtype group were detected on farms of every production type. The presence of HEV strains belonging to different subtype groups was detected only in fattening pigs, in which co‐circulation of 3f and 3e was observed in animals housed in two fattening units, whereas 3f and 3c strains were found in one unit.

**Table 4:** HEV strains circulating in pig farms in particular Polish provinces.

Virus subtype	Farm type	Province	Number of strains	Total
3c	Farrow‐to‐finish	Mazovia	6	6
	Fattening unit	Kujawy‐Pomerania	3	3
				
3i	Farrow‐to‐finish	Mazovia	2	29
		Podlasie	13	
		Warmia‐Masuria	14	
				
3e	Farrow‐to‐finish	Mazovia	11	12
		Warmia‐Masuria	1	
				
	Fattening unit	Lublin	8	16
		Mazovia	8	
				
3f	Farrow‐to‐finish	Podlasie	3	3
				
	Fattening unit	Kujawy‐Pomerania	9	46
		Lublin	1	
		Łódź	16	
		Mazovia	19	
		Opole	1	
				
3^a^	Farrow‐to‐finish	Warmia‐Masuria	1	1
				
	Fattening unit	Kujawy‐Pomerania	1	10
		Łódź	2	
		Mazovia	7	

^a^Unassigned virus subtype.

### 3.4. Nucleotide Sequence Similarity and Phylogenetic Analyses of the HEV Strains

The similarity of the nucleotide sequences of the ORF2 genome fragment of pig HEV‐3e strains detected in the study ranged from 87.4% to 100%. In the case of 3i strains, it was from 90.9% to 100% and for 3f strains, from 86.8% to 100%. At least 95.1% sequence similarity was observed for the group of 3c strains. HEV‐3c, 3f and 3i strains with 100% sequence concordance within each subtype group were only found on the same farm. However, identical HEV‐3e strains were not only detected on the same farm but also on farms from geographically distinct locations due to possible pig movement between farms of different production types. Genetic variability between virus strains of a particular subtype was also observed between farms of different production types.

**Figure 4:**
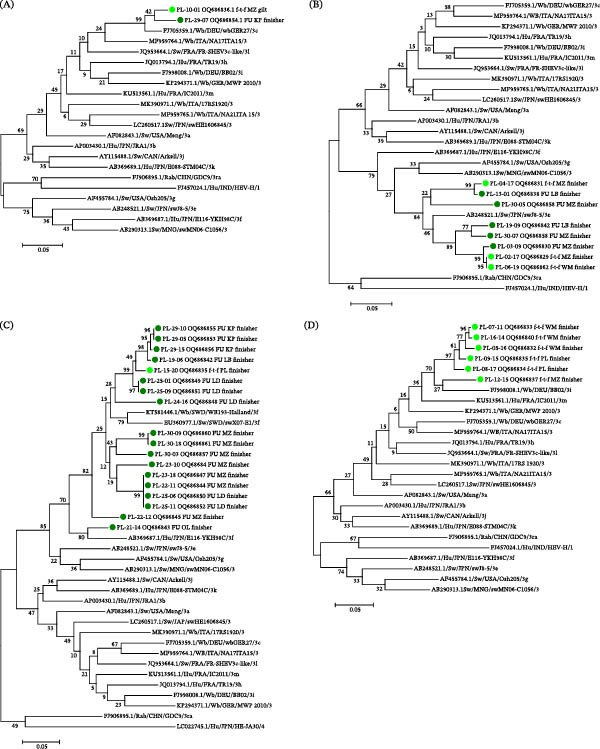
The phylogenetic relationship of 3c (A), 3e (B), 3f (C) and 3i (D) HEV strains circulating in pigs in Poland. Pig HEV strains from Poland detected in f‐t‐f and FU farms are marked on the trees with symbols of light and dark green circles, respectively. The phylogenetic tree was constructed using the nucleotide sequences of the ORF2 genome fragment (493 bp) of HEV gt 3 strains. The sequences of human HEV‐1 (FJ457024) or HEV‐4 (LC022745) strains were used as outgroups.

HEV‐3i strains detected on f‐t‐f farms formed two closely related clusters encompassing strains from WM and PL, as well as from the MZ province, respectively. Likewise, 3c strains were assigned to a single clade despite their originating from farms of different production profiles (f‐t‐f and FU) from geographically distinct locations. The phylogenetically related clades were also formed by 3e strains detected in pigs from farms located in the neighbouring provinces. Interestingly, the strains from FU farms in MZ were not clustered together but into different virus groups gathering strains detected on farms in LB or WM provinces. As with 3e strains, a similar pattern of strain distribution on the phylogenetic tree was seen for 3f viruses found on FU farms from different regions (Figure 4).

## 4. Discussion

HEV infections in pigs are caused by zoonotic virus strains and are characterised by an asymptomatic course. Although infections do not result in animals’ deaths or breeding losses, they have a direct impact on public health related to food products derived from infected animals entering the food chain. Therefore, food of animal origin plays a significant role in the aetiology of viral foodborne infections in humans [11, 48]. In the European population of pigs, HEV‐3 is detected, although single cases of infections caused by HEV‐4 strains have also been reported [9, 49]. HEV‐3 infections occur in pigs worldwide, and regional differences in infection prevalence resulting from different ages or production stages of tested animals have been observed [30, 49, 50]. Infections in pigs occur commonly when assessment is based on the detection of specific anti‐HEV antibodies. For instance, in Europe, the highest seroprevalence rates have been found among fattening pigs (30.8%–75.8%) [51, 52] and sows (59%–80%) [53]. Of note is that in Asia, the HEV seroprevalence in sows was even higher (82.5%) than that in Europe [54]. Among factors which can influence HEV spread in a pig herd are animal age (<3 months) and farm type. A higher frequency of HEV infection has been observed in animals from organic farms than in those from closed‐cycle or free‐range farms [55]. As seen in previous European studies, HEV was highly prevalent in fattening pigs [18, 22, 24, 51]. In this study, pigs were bred on three types of farms: farrow‐to‐finish, farrow‐to‐wean and fattening. Animals from these operations, which are representative of pig production in Poland, were tested for HEV RNA. Fattening pigs constituted the largest group of studied animals, and among them, 35.9% infection prevalence was recorded.

A higher percentage of HEV‐positive animals was observed among fatteners from farrow‐to‐finish farms than among these pigs from fattening units; farrow‐to‐finish fatteners were 43.6% positive and fattening unit fatteners were 32.3% positive. Similar rates of HEV detection have been found in 3–6‐month‐old fattening pigs raised in other European countries [17, 20, 22, 23, 56–60]. Of note is that a low infection rate of <15% has been recorded in Belgium, Bulgaria, Slovakia and Corsica [9, 19, 21, 26]. A contrastingly high infection rate to that in our observations was the rate of almost 65% of tested animals from this age group and production type, which was detected in Italy [61]. In gilts and sows tested in Poland, HEV was only detected in animals from farrow‐to‐finish farms and not in pigs from farrow‐to‐wean farms. There are scarce scientific data on the HEV occurrence in gilts. It appears that gilt HEV infections can occur infrequently in Poland as only 2.9% of pooled faecal samples collected from gilts were positive for HEV RNA. Likewise, a low virus prevalence in gilts not exceeding 0.4% has been observed on farrow‐to‐finish farms in Bulgaria [26]. Of note is that the weaner pigs, in contrast to older animals, more frequently acquire HEV infections due to a loss of their maternal immunity; therefore, the low prevalence (1.09%) of virus‐positive sows from Poland may not be surprising. A higher protection of sows usually results from the acquired strong immunity developed during repeated contact with the virus. Thus far, a similar infection prevalence (1.6%) in sows has also been observed in Bulgaria [26]. However, there is also evidence that in the European population of sows, the level of HEV infections has far exceeded this, at a maximum of 15% [62]. Among the risk factors associated with HEV infection is the pig breed. Animals of Sino‐European and Landrace, Duroc and Large White mixed breeds were indicated as genetic types which might be more susceptible to virus infection than Landrace × Large White [63]. However, this particular factor cannot be linked with any results of HEV prevalence in pigs studied in the present research in Poland, as similar infection rates between 20% and 27.8% were observed in animals representing different breeds. Multiple virus genotypes circulate in most world regions and prevail in different patterns geographically [10]. However, in Europe, the broad heterogeneity of the circulating HEV‐3 subtypes has been detected in pigs with predominating 3e, 3f and 3c virus strains. Interestingly, geographical subtype distribution has been observed. For instance, 3c subtypes were mainly detected in Northern Europe (Germany, the Netherlands), while 3e and 3f were detected in Central Europe (Czech Republic and Poland) [30]. As in several European countries, HEV‐3f strains also predominate in pigs in Poland. Furthermore, they were also found in Poland’s neighbours, Germany and the Czech Republic. Of note is that Germany is the second‐largest exporter of pigs to Poland after Denmark; therefore, trade of live animals could facilitate virus transmission and its geographical spread. Likewise, pigs are also imported to Poland from the Czech Republic, where 3f strains predominate in pigs. It has been previously shown that they gather in closely related phylogenetic clusters with Polish 3f pig strains [30]. The 3i, 3e and 3c strains were also detected in farmed pigs in Poland and noted to circulate in pig herds in Europe [10, 30, 64]. HEV‐3i strains were the majority of strains detected in pigs in Slovakia, but in Poland, they only constituted the second largest group circulating in this animal species. Contrastingly, to the 3f dominance among the strains detected in Polish pigs, 3i strains were the most commonly found in the population of Polish wild boars [34]. Infections with 3i strains have been reported in both pigs and wild boars in the same provinces (PL and WM) in north‐eastern Poland, as well as in these species in neighbouring Lithuania [65]. Regardless of their geographical origin, HEV‐3i strains from Poland revealed high (91.8%– 93.3%) nucleotide sequence similarity, indicating a possible cross‐species virus transmission. It appears that within the group of pig 3c strains, closely related strains originating from Poland and Germany, Poland and the Netherlands, as well as Germany and the Netherlands circulate. This most likely results from the animal trade between these countries. The 3c strains circulating regionally on the farms also showed a high degree of phylogenetic similarity. Likewise, the regional circulation of HEV strains between fattening farms has previously been shown in Germany [50]. Interestingly, in the case of 3e and 3f strains, their genetic diversity was observed not only among strains detected in different Polish provinces but also among strains found on the same farm. The circulation of HEV‐3 strains of the same subtype in pigs and wild boars has also been observed more widely in Europe [66, 67]. Nevertheless, a 100% nucleotide sequence similarity has only been seen so far in Italian 3c strains detected in pigs and wild boars from the same geographic region. This finding suggests an interspecies transmission of HEV‐3 strains between closely related animal species [68]. This study has several limitations which are related to: (i) difficulties in identification of the virus subtypes for all detected virus strains, (ii) testing of pooled instead of individual faecal samples and (iii) a possible underestimation of virus occurrence in fattening pigs raised on different farm types due to a low number of farms of each production type covered by monitoring in particular Polish provinces. In an effort to acquire a better understanding of HEV subtype occurrence in fattening pigs and to recognise the usual appearance of virus strains of unknown subtype detected in pigs, the additional amplification and sequencing of a smaller, 348‐bp fragment of the ORF2 virus genome were performed (results not shown in this study). Unfortunately, even this approach did not make successful subtype identification of all detected virus strains possible, and some strains remained unassigned to any currently known virus subtypes. Another possible limitation could be associated with the sampling scheme, which only covered a low number of farms representing a particular production type (fattening unit, farrow‐to‐finish and farrow‐to‐wean) in the country. To avoid interpretation bias in the assessment of infection prevalence in pigs, a reasonably large number of pooled faecal samples from each farm were taken for analysis. It should also noted that testing of pooled rather than individual faecal samples could have an impact on the results obtained on infection prevalence in pigs. Nevertheless, compared to testing of individual samples, the collection of pooled faeces allowed analysis of a larger and more representative number of animals at the farm level. It was particularly important for pig commercial farms breeding even several thousands of animals. Likewise, this approach made possible detection of infected animals present on the farms, characterised by a low level of infection rate. Ultimately, this approach could still provide reliable data on infection prevalence and distribution of HEV subtypes in different animal groups at least at the farm level. Certainly, if a higher number of farms representing a particular method of breeding were monitored, then data regarding virus infections in pigs would be more accurate. On the other hand, testing of fattening pigs from farrow‐to‐finish and fattening farms from regions with intensive pig farming can be considered a sentinel study and gives an overview of the occurrence of HEV infections on these farm types in Poland. Besides different factors having an influence on the level of infection prevalence in a particular population of pigs, the husbandry practices, including how manure is managed, could also have an impact on the results obtained. However, the influence of operational procedures on the farm epidemiology of HEV was not covered by this study. Ultimately, the strict biosecurity measures introduced on Polish farms to protect herds from ASF infections could have a cross‐protective effect on the number of infections caused by different viral pathogens, including HEV [69].

## 5. Conclusions

This is the first report describing the detection of HEV‐3 infections on commercial pig farms of different production types in Poland. As sentinel study results, the results indicate that HEV infections were predominantly found in fattening pigs; farm type and geographical location did not emerge as significant factors, although the sample size and distribution limit definitive conclusions about these variables. The virus strains circulating in animals represent virus subtypes previously identified in the European population of pigs with a predominance of 3f virus strains. In this light, virus detection in fattening pigs could pose a food‐related risk of HEV infection when the pigs reach slaughter age.

## Author Contributions

**Iwona Kozyra**: methodology, investigation, data analysis, writing – original draft. **Justyna Joniec-Wiechetek**: writing – original draft. **Jacek Żmudzki**: methodology, writing – review and editing, funding acquisition. **Artur Rzeżutka**: conceptualisation, methodology, data analysis, writing – original draft, writing – review and editing, supervision.

## Funding

This project has received funding from the European Union’s Horizon 2020 Research and Innovation Programme under Grant Agreement (Grant 773830) in the One Health European Joint Programme. The National Veterinary Research Institute was co‐financed from funds for science in the years 2018–2022 allocated for the implementation of an international co‐financed project by the Ministry of Science and Higher Education of the Republic of Poland.

## Ethics Statement

This study did not require the approval of an Ethics Committee as it did not involve animal experiments. Freshly voided faeces were collected from the pen’s ground during the routine veterinary visits, which have been conducted with adherence to the international guidelines for animal care and welfare. During this study, no clinical intervention or animal examination were conducted by vets collecting samples. Sample collection did not violate animal welfare laws. The animal owners provided informed consent to participate in this study.

## Conflicts of Interest

The authors declare no conflicts of interest.

## Data Availability

The nucleotide sequences of the HEV strains were deposited in the GenBank database at https://www.ncbi.nlm.nih.gov/genbank/ under Accession Numbers OQ686829–OQ686862.
